# The impact of pandemic-related social distancing regulations on exercise performance—Objective data and training recommendations to mitigate losses in physical fitness

**DOI:** 10.3389/fpubh.2023.1099392

**Published:** 2023-02-28

**Authors:** Tania Zieschang, Fabian Otto-Sobotka, Abdul Shakoor, Sandra Lau, Michel Hackbarth, Jessica Koschate

**Affiliations:** ^1^Geriatric Medicine, Department for Health Services Research, School of Medicine and Health Sciences, Carl von Ossietzky University Oldenburg, Oldenburg, Germany; ^2^Epidemiology and Biometry, Department of Human Medicine, School of Medicine and Health Sciences, Carl von Ossietzky University Oldenburg, Oldenburg, Germany; ^3^Department of Cardiology, Erasmus Medical Center Rotterdam, Rotterdam, Netherlands

**Keywords:** social distancing, aged, exercise training, physical fitness, physical activity, health promotion, COVID-19 pandemic

## Abstract

**Introduction:**

In the context of the COVID-19 pandemic in Germany, governmental restrictions led to the closure of sports facilities for several months. To date, only subjective and fitness-tracking related data on physical activity during the pandemic are available. Using data of a chip-controlled fitness circuit, training data as a measure of physical performance before and after the lockdown during the first wave of the COVID-19 pandemic will show the impact of the training interruption on exercise performance in middle-aged and older adults. The re-training data are analyzed, to extract practical recommendations.

**Methods:**

Objective training data of 17,450 participants [11,097 middle-aged (45–64 yrs), 6,353 older (≥65 yrs)] were exported from chip-controlled milon^®^ fitness circuit systems before and after the first COVID-19 related lockdown in Germany. The change in the product of training weight (sum of lifting and lowering the training weight) and repetitions on the leg extension resistance exercise device (leg score) between the last three training sessions before the lockdown and the first ten training sessions after individual training resumption as well as the last training session before the second lockdown in October 2020 was analyzed.

**Results:**

Participants who trained with high intensity before the lockdown, experienced deleterious effects of the training interruption (middle-aged group: −218 kg, older group: ~−230.8 kg; *p* < 0.001 for change in leg score from to post-lockdown) with no age effect. Participants training with a leg score of more than 3,000 kg did not resume their leg score until the second lockdown.

**Conclusion:**

The interruption of training in a fitness circuit with combined resistance and endurance training due to the lockdown affected mainly those participants who trained at high intensity. Apparently, high-intensity training could not be compensated by home-based training or outdoor activities. Concepts for high-intensity resistance training during closure of sports facilities are needed to be prepared for future periods of high incidence rates of infectious diseases, while especially vulnerable people feel uncomfortable to visit sports facilities.

**Trial registration:**

Identifier, DRKS00022433.

## 1. Introduction

The first wave of the COVID-19 pandemic had a great impact on the health care system and the social life in Germany. Since no vaccines were available in 2020, a high number of patients with SARS-CoV-2 infections suffered from severe courses, which led to an overload of the health care system, especially in Intensive Care Units. Until 2023, about 37.5 Mio. cases were reported during several COVID-19 waves in Germany, which were caused by different mutations of the virus (1, 2). To reduce the rate of infection during the first wave of the COVID-19 pandemic, the German government enacted a hard lockdown from March to June 2020, which included not only the temporary closure of retail shops, cultural events, and restaurants, but also all sports facilities.

Due to their high risk for severe courses of a COVID-19 infection, older adults voluntarily limited their life space and physical activity substantially to protect themselves from infection (3). As physical activity is of high importance to promote health, wellbeing, physical fitness, and participation in older people (4–11), short- and long-term side effects of the restrictions are of great concern.

Physical activity decreased during the pandemic-related lockdown, especially in formerly very active adults, in many countries (8, 12–14). As known from bed rest, step reduction, and detraining studies, recovery of muscle mass and aerobic capacity, which are reduced by inactivity, require a longer period of time in older, compared with younger adults (15–19). Low skeletal muscle mass is well-documented as a cause of diverse negative outcomes, such as functional impairment and morbidity in older adults (5, 20).

To date, only self-reported (8, 12, 13, 21) and activity tracking data (14) on changes in individual physical activity and exercise behavior during the pandemic were published. Chip-controlled fitness circuits give access to objective measures of exercise training performance, which we postulate as a surrogate of physical fitness, before the lockdown and after training resumption.

The aim of this observational study is to describe how individual exercise training performance on resistive and aerobic exercise training devices changed following the first lockdown period in different age groups and in respect to baseline training intensity. Further, predictive factors for a pronounced decline, as well as factors enhancing the recovery of exercise training performance after the lockdown will be identified in regression models.

## 2. Material and methods

### 2.1. Study design and data base

This observational study uses data from the milon^®^ fitness circuit system (milon industries, Emersacker, Germany). Apart from age and gender of the participant, the system stores the following training data: workout weight in concentric, eccentric, and adaptive mode, as well as while lifting and lowering the weight, the number of repetitions per set, the number of sets, as well as the duration of the training activity on each device. On the ergometric devices, power and training duration are documented. If an appropriate belt is worn, heart rate (HR) is also registered. A regular training in a milon^®^ fitness circuit includes 60 s for each resistance exercise, 30 s time intervals to change between two devices and 240 s for the endurance training devices. Sets can be repeated as desired. In this analysis, only data from the leg extension, the seated row devices for resistance training of a larger upper-body muscle group, and the cycle ergometers were included.

Data of participants with a training registration during the 4 weeks before the lockdown were exported anonymously from the milon^®^ care cloud, owned by milon industries, in consideration of the general data protection regulation of the European Union. The data analysis was approved by the ethics committee of the medical faculty at the Carl von Ossietzky University of Oldenburg before the beginning of data extraction (2020-061) and the study was registered at the German clinical trials registry (DRKS00022433).

### 2.2. Study population

Overall, data sets of initially 43,843 participants above the age of 45 years with training data in February 2020 (1 month before the first lockdown) were exported until October 2020 from the milon^®^ care cloud. The participants were divided into a “middle-aged” (45–64 yrs) and an “older” (≥65 yrs) age group.

### 2.3. Outcomes

For the resistive exercise devices, training performance was measured with a score, calculated from the product of training weight (sum of weight for lifting and lowering) and overall number of repetitions during one training session (leg score, rowing score). The scores reflect the total weight lifted per training session and allow a comparison in spite of different training habits (low training weight, many repetitions vs. high training weight, few repetitions). For the endurance exercise data, we calculated an endurance score [HR divided by work rate (WR)] to account for a higher HR when performing the same WR, which indicates lower physical capacities.

The changes for the leg score were the primary outcome of the analysis. Secondary outcomes were changes in the rowing and the endurance scores.

### 2.4. Data analysis

As measure for baseline training performance, scores were averaged from the last up to three training sessions in February 2020, as available. Three training intensity groups (TIG; low, moderate, high) were determined, applying the tertials of the leg score before the first lockdown (pre-COVID-19_legscore_) as cut-off values. The time course of the changes of the leg, rowing and endurance scores before the lockdown are described for the first ten individual training sessions after the re-opening of the fitness circuits to show potential losses of performance. To assess the progression of recovery, the last session before the second lockdown (starting on November 2nd in 2020) was included as well. Only participants with available data for the leg extension device for the described time points, as well as individuals who additionally trained with the rowing resistive exercise device and a cycle ergometer, were included in the analysis. The first registered training was used to calculate the individual training interruption in days. In the generalized, additive regression models all available training sessions from February to October 2020 were included.

### 2.5. Statistical analysis

A mixed linear regression model, including gender (male, female), age group (older, middle-aged), training session (session 1–10 after the lockdown) and TIG (low, moderate, high) was used to describe the changes in the leg, rowing and endurance scores, before and after the lockdown.

In addition, three generalized additive regression models were constructed to predict the difference between the pre-COVID-19_score_ and the first post lockdown training score for the leg, rowing, and endurance scores. The set of covariates was gender, age (in years), average training weight (sum of weight lifted and released), duration of training set, average number of repetitions in a set, number of training sets, pre-COVID-19_score_ for leg score, rowing score, endurance score, and number of days paused. The variable selection was performed stepwise backwards using the Akaike Information Criterion (AIC).

The increase of the leg score, as the most important indicator for the weight bearing muscles, after training resumption relative to the pre-COVID-19_score_, was modeled in a generalized additive mixed regression. All post lockdown trainings were included as repeated measurements and the number of completed trainings was included as a random effect per person. For the fixed effects, the same set of covariates as in the previous models was eligible, plus the number of completed trainings. Variable selection was again performed with a stepwise backwards algorithm using the AIC. Further, we included pairwise interaction terms of average training weight and average number of repetitions with the number of completed trainings. All metric variables and interactions were modeled with the possibility for a flexible non-linear effect using a P-spline basis and a tensor product P-spline, respectively.

For all regression models, the regression coefficients with 95% confidence intervals are reported as well as the adjusted R^2^. For the non-linear effects, the estimated centered effect curves are plotted and for the bivariate interaction terms the estimated effects are provided in heatmaps with contour lines. Calculations were performed using the software R 4.1.0 (22) with the package “mgcv” (23).

## 3. Results

### 3.1. Changes in exercise training performance after the lockdown

**Figure 1:**
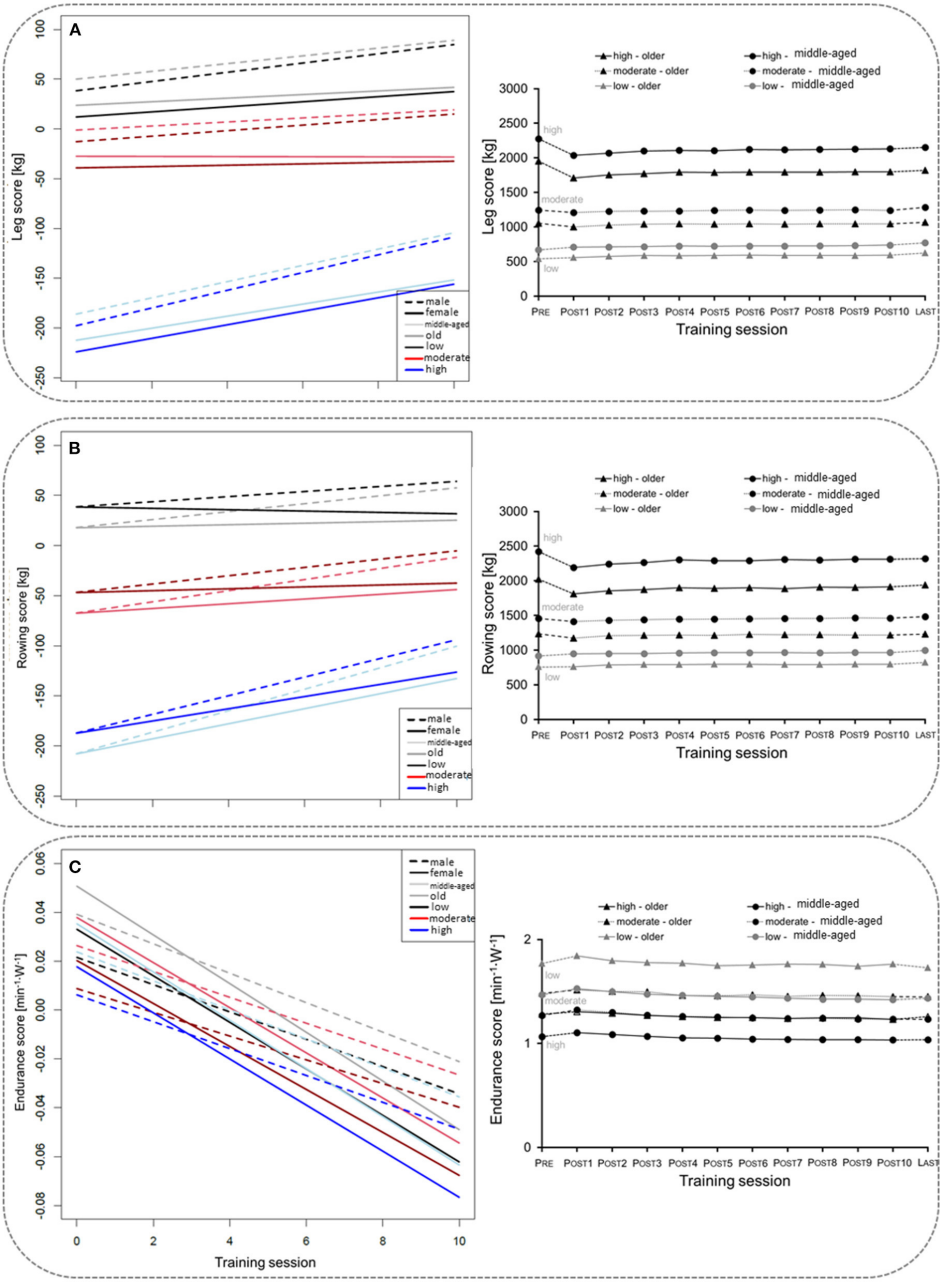
Left column: Course of changes in the for the leg score **(A)**, rowing score **(B)**, and endurance score **(C)** over the first ten training sessions after the lockdown. Negative values indicate losses in the leg and rowing score in comparison to the values before the lockdown, and lower heart rates per given work rate. Right column: Description of the mean for the leg score **(A)**, rowing score **(B)**, and endurance score **(C)** before (Pre), during the ten first sessions after the lockdown period 1(Post1–10), and for the last training session (Last) before the second lockdown in Germany, considering age and training intensity groups (TIG; low, moderate, high) based on the leg score before the lockdown (pre-COVID-19_legscore_). SE are too small to be presentable.

Overall, 17,450 participants (11,097 middle-aged, 6,353 older age group) with complete data sets (pre, post 1–10) were included in the mixed linear regression model. The participants' baseline performance and cut-off values between the low, the moderate, and the high TIG in each age group are shown in Table 1. The time courses of the leg, rowing, and endurance scores over the first wave of the COVD-19 pandemic are shown descriptively for each age group and TIG in Figure 1 (right column). In the left column of Figure 1 and in Table 2, the results of the mixed linear regression model to evaluate the influence of the factors gender, age, TIG and training session on the change in leg, rowing and endurance scores during the first 10 sessions after the lockdown are shown. A substantial decrease in the leg and rowing score is exclusively seen in the high TIG in both age groups. In both scores a complete recovery to the pre-lockdown performance was not reached throughout the first ten training sessions in the high TIG. As expected, performance was lower in the older age group (opaque lines).

**Table 1:** Participants' characteristics by age groups and training intensity groups.

**Age group**	**Pre-COVID-19_Legscore_ mean ±SD (median) [kg]**	**Rowing score mean ±SD (median) [kg]**	**Endurance score mean ±SD (median) [min^−1^·W^−1^]**	**TIG according to pre-COVID-19_Legscore_ [kg]**	**Cut off values [kg] to define TIGs**	***n***	**Gender**		**Age [years] mean ±SD**	**Training break [days] mean ±SD**
							**Female [*****n*** **(%)]**	**Male [*****n*** **(%)]**		
Middle-aged *n* = 11,097	1,395.14 ± 830.16 (1,237.67)	1,601.61 ± 947.73 (1,426.00)	1.26 ± 0.48 (1.16)*	Low	< 976.00	3,701	3,050 (17.5%)	651 (3.7%)	56 ± 5	84 ± 31
				Moderate	976.00–1,548.22	3,697	2,558 (14.7%)	1,139 (6.5%)	55 ± 5	84 ± 32
				High	>1,548.22	3,699	1,591 (9.1%)	2,108 (12.1%)	55 ± 5	85 ± 32
Older *n* = 6,353	1,182.22 ± 716.90 (1,050.00)	1,343.33 ± 828.73 (1,190.33)	1.49 ± 0.67 (1.34)^#^	Low	< 816.00	2,124	1,502 (8.6%)	622 (3.6%)	73 ± 6	86 ± 31
				Moderate	816.00–1,324.33	2,110	1,209 (6.9%)	901 (5.2%)	72 ± 5	85 ± 32
				High	>1,324.33	2,119	783 (4.5%)	1,336 (7.7%)	71 ± 5	85 ± 32

TIG, training intensity group; SD, standard deviation, ^*^n = 5,444, ^#^n = 2,696 (numbers reduced for endurance score as only 49%, respectively 42% of participants wore the belt to measure the heart rate.

**Table 2:** Mixed regression analysis for the first 10 training session.

	**Leg score**			**Rowing score**			**Endurance score**		
	**Estimate [kg]**	**SE**	**Sig**.	**Estimate [kg]**	**SE**	**Sig**.	**Estimate [min**^−1^·**W**^−1^**]**	**SE**	**Sig**.
Intercept	22.1	11.379	0.052	17.8	12.5	0.157	0.051	0.008	< 0.001
Gender (male)	36.6	11.1579	0.001	0.2	14.5	0.988	−0.011	0.009	0.210
Older adults	−12.8	12.2801	0.299	0.8	1.3	0.561	−0.010	0.001	< 0.001
Training session	2.1	1.1181	0.057	20.7	13.9	0.136	−0.018	0.009	0.048
TIG moderate	−52.6	14.434	< 0.001	−85.4	16.3	< 0.001	−0.013	0.010	0.221
TIG high	−240.1	15.1022	< 0.001	−225.8	17.1	< 0.001	−0.015	0.011	0.167
Older adults training session	1.0	1.207	0.422	3.2	1.5	0.032	0.004	0.001	< 0.001
TIG moderate training session	−1.6	1.4212	0.267	−1.4	1.4	0.319	0.000	0.001	0.656
TIG high training session	5.1	1.4296	< 0.001	1.6	1.7	0.337	0.001	0.001	0.529

The intercept value describes the change after the lockdown for a middle-aged, female adult in the low training intensity group (TIG).

In the regression models to model influencing training habits before the lockdown for respective losses in the leg and rowing scores after the lockdown, negative values (age, the average number of sets, and the duration of the training pause) imply higher losses from pre- to post-lockdown (Table 3). The applied training weight and number of repetitions appear to reduce losses, however, in the model this is only true for training at very low intensities. After passing a certain threshold, the equation is inversed with higher intensity training, as characterized by a high number of sets, resulting in higher losses compared to baseline. The endurance score is mainly unchanged (Table 3).

**Table 3:** Results of the linear regression models to predict losses in the leg, rowing, and endurance scores from pre- to post-lockdown and the generalized additive mixed regression model to predict the regain of the leg score after individual training resumption.

	**Prediction of losses from pre- to post-lockdown**							**Prediction of increases after the lockdown**		
	**Leg score (*****R**^2^* = **0.157)**		**Rowing score (*****R**^2^* = **0.167)**		**Endurance score (*****R**^2^* = **0.030)**			**Post-lockdown increase leg score (*****R**^2^* = **0.663)**		
***n*** = **23,299**	**Estimate [kg]**	**CI 95% [kg]**	**Estimate [kg]**	**CI 95% [kg]**	***n***= **12,174**	**Estimate [kg]**	**CI 95% [kg]**	***n*** = **69,197**	**Estimate [kg]**	**CI 95% [kg]**
								Intercept [kg]	−598.1	−619.7–−576.5
Gender (male)	22.1	8.7–35.4	67.5	52.0–83.0	Gender (male)	−0.041	−0.056–−0.025	Gender (male)	0.5	−6.8–7.8
Age [years]	−3.2	−3.8–−2.6	−4.1	−4.7–−3.4	Age [decade]	0.007	−0.008–0.009	Age [years]	−0.8	−1.2–0.5
Avg training weight [kg]	5.6	5.0–6.1	5.1	4.5–5.7	Duration [minutes]	0.009	−0.011–0.011	Sets^#^ = 2 [number]	655.3	652.2–658.4
Avg repetitions [number]	13.0	11.4–14.5	14.8	13.0–16.6	Avg heart rate [min^−1^]	0.000	−0.001–0.000	Sets^#^ = 3 [number]	1,244.9	1,240.5–1,249.3
Sets^*^= 2 [number]	−9.0	−26.3–8.2	−4.0	−23.5–15.5	Avg rpm [min^−1^]	0.002	−0.003–−0.002	Sets^#^ = 4+ [number]	1,751.7	1741.7–1761.7
Sets^*^= 3 [number]	−93.3	−124.1–−62.5	−100.3	−136.6–−64.0	Sets [number]	−0.035	−0.079–0.008			
Sets^*^= 4 [number]	−219.9	−289.6–−150.2	−158.9	−223.2–−94.6						
Sets^*^= 5+ [number]	−273.4	−402.3–−144.6	−233.1	−366.4–−99.8						
Pre-COVID-19_legscore_ [kg]	−0.3	−0.4–−0.3	0.0	0.0–0.0	Pre-COVID-19_legscore_ [kg]	−0.002	−0.001–0.001			
Pre-COVID-19_rowscore_ [kg]	0.0	−0.0–0.0	−0.4	−0.4–−0.3	Pre-COVID-19_endurancescore_ [min^−1^·W^−1^]	−0.101	−0.114–−0.088			
Training pause [days]	−0.8	−0.9–−0.7	−0.9	−1.1–−0.8						

Regression coefficients with 95% confidence intervals.

Avg, average over all sets of the last three training sessions; rpm, revolutions per minute; pre-COVID-19_legscore_, pre-COVID-19_rowingscore_, pre-COVID-19_endurancescore_, average of the three last training sessions before the lock down for the leg extension device, the rowing exercise device, the cycle ergometer, respectively. Sets, the number of times a participant repeated an exercise. ^*^Median number of sets from the last three training sessions before the lockdown, ^#^number of sets per training session after the lockdown until October 31st 2020. Negative values indicate a higher loss in the particular score, positive values indicate a reduced loss.

### 3.2. Prediction of training recovery

**Figure 2:**
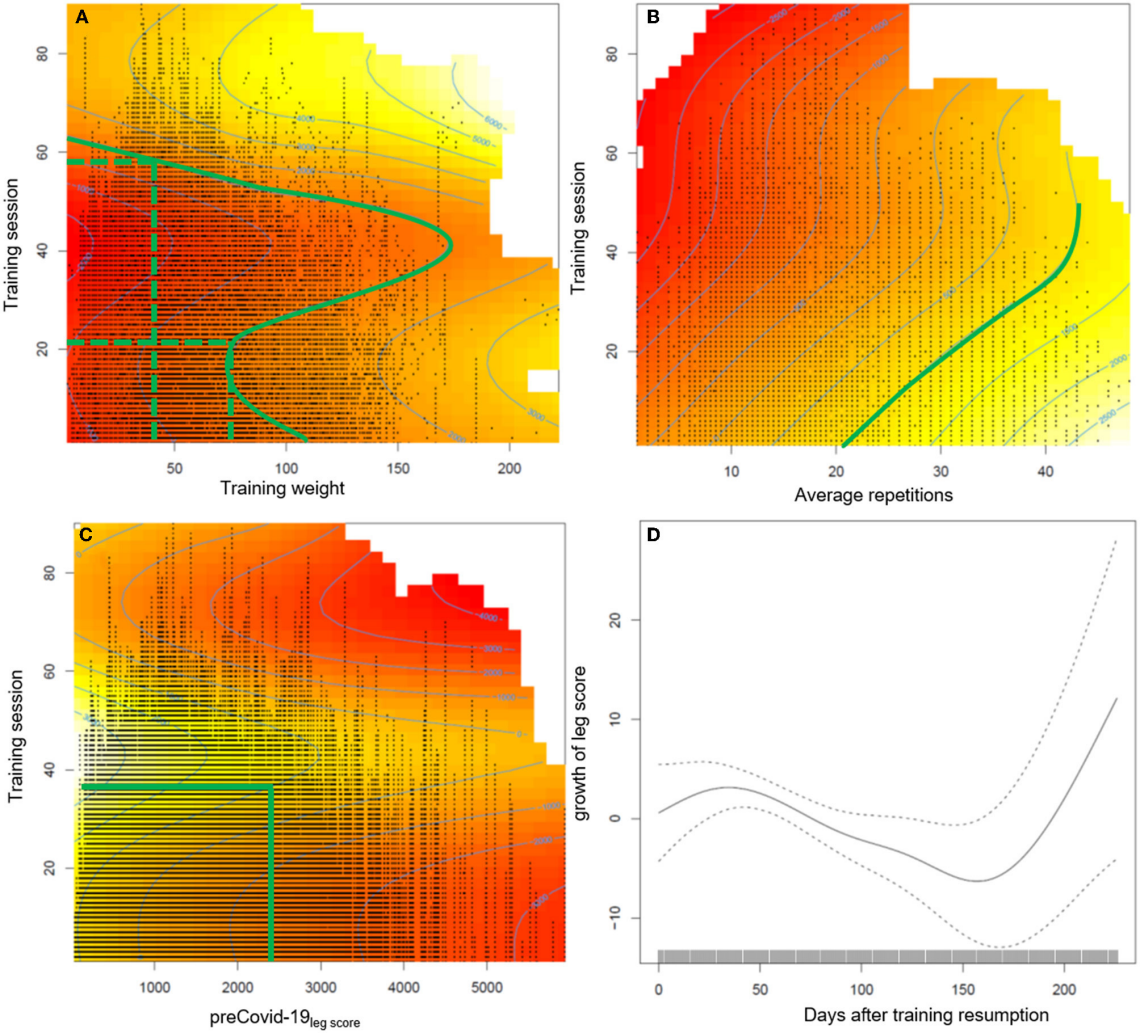
Presentation of the estimated effects on the increase of the leg score of **(A)** average training weight per repetition (sum of lifting and lowering the training weight), **(B)** average repetitions per set, **(C)** pre-COVID-19_legscore_, and **(D)** the number of days after training resumption during the post lockdown training sessions. The heat maps include contour lines, which denote increase or loss in the leg score compared to the first training session after the lockdown. As the intercept of the model is −598.06 kg, the zero line still indicates a loss in training performance of at least ≈600 kg compared with baseline level, the difference will be larger depending on age and gender. Therefore, we marked the contour line 1,000 in **(A, B)** which indicates recovery of baseline values. The red color indicates a permanently reduced leg score until October 2020.

The generalized additive mixed regression model, predicting the increase of the leg score after individual training resumption post lockdown, reveals that the number of sets a participant carries out, and to a small degree also male gender, predict an accelerated increase of performance, e.g., two instead of one set of repetitions led to ~100% more increase in the leg score (~650 kg) (Table 3). Age had only a small negative effect (Table 3). The effects of different training regimens, that participants applied when they resumed training after the lockdown, on their individual progress in regaining training performance are shown in Figure 2. The contour lines and colors show the course of the increase or reduction in the leg score. The visualization with heat maps allows the identification of effective training strategies. To clarify, we give some examples of the information the heat maps contain: as indicated by the dashed green lines in Figure 2A, participants training with an average training weight of 75 kg (sum of lifting and lowering) require about 20 training sessions to regain their exercise performance before the lockdown, while someone training with ~ 40 kg would need ~60 sessions. On the leg extension device, at least 15–20 repetitions should be performed per set; the more repetitions, the greater the benefit (green line in Figure 2B). Figures 1, 2 consistently show, that participants with lower pre-COVID-19_legscores_ needed fewer training sessions to regain their baseline levels (Figure 2C). Participants with pre-COVID-19_legscores_ higher than ~3,000 kg did not reach their baseline levels by the end of October 2020. The mere number of days after training resumption, irrespective of training sessions, did not predict the increase of the leg score (Figure 2D).

## 4. Discussion

The aim of this data analysis was to show, (i) how individual exercise training performance, which we interpret as a surrogate of physical fitness, changed due to the training interruption caused by the first COVID-19 lockdown in different age groups with different initial training intensities, and (ii) to identify training regimens beneficial for a fast recovery of the baseline training performance.

### 4.1. Exercise training performance after the lockdown

Interestingly, we see similar results for both age groups: Participants who trained with high intensity before the lockdown had pronounced and long-lasting reductions in training weights for lower (leg score, Figure 1A) and upper body (rowing score, Figure 1B) exercise. For cycle ergometer exercise, the most pronounced increase in HR per given work rate was about 0.05 min^−1^·W^−1^, thus 2.5 beats·min^−1^ for 50 W, in the middle-aged adults of the low TIG. Physiological and clinical relevance is questionable. For comparison, differences of about 10 beats·min^−1^ at a WR of ~50 W after 60 d of bed rest were reported (24). The regression models confirmed the result showing a substantial reduction in performance in participants who trained with a higher number of sets before the lockdown (Table 3). Accordingly, Suzuki et al. (8) reported a greater decrease in self-reported physical activity in people who were highly active before the COVID-19 pandemic. This finding is of substantial relevance, as the main concern of health care professionals and politics was on the most vulnerable groups, the frail older population. However, adverse effects of the protective social distancing regulations appear to have affected the physical capacities of the fittest seniors the most.

The losses for the leg, and especially the rowing score were lower in male compared with female participants. In contrast, in a review no sex-dependent differences in submaximal strength after training cessations of up to 200 days were reported (18), and natural age-related loss in muscle power appears to be lower in women (−1.7 vs. −3% per year) (25). With no information about physical activity during the lockdown period, it can only be speculated, that male individuals integrated more upper body muscular exercise in their daily life.

The factor age had small influence on the losses in performance and only marginal impact during the recovery phase. Generally, longer recovery periods after phases of inactivity, due to bed rest or detraining phases have been reported for older people several times in the literature (15–19). In a recent review, analyzing short term (4–14 d) immobilization studies, comparing older and younger adults, greater muscle atrophy was not a consistent finding for older adults, and high-quality research on this topic is lacking (26). The individual level of physical activity might be more important for muscle atrophy than chronological age. Kirwan et al. (5) consider the “catabolic crisis” model, as proposed by English and Paddon-Jones (27), to facilitate sarcopenia during rather short phases of inactivity and not as a gradual process. Individual trajectories of declines in physical performance over the course of life seem to be variable and task specific (28–30). Considering the catabolic crisis model, the lockdown-induced training cessation might have long-lasting effects on the high intensity group, as sarcopenia is well-known as a main influencing factor on frailty (31). Furthermore, compared to values of the same time interval for 2019, an increased fall rate was reported for older adults after the lockdown period in 2020 (32). We conclude, that the increased offers for guidance on physical activity and online exercise classes (13) or the individual exercise routines, encompassing walking and cycling, seem to be sufficient to enable uptake of the pre-lockdown exercise training level after longer interruption for the low and moderate exercise groups, but not for the high intensity training group.

### 4.2. Prediction of training recovery

The semiparametric regression model revealed that to regain the pre-COVID-19_legscore_, 20–60 sessions were necessary, with fewer sessions required when training with a higher weight on the leg extension training device. Further, at least 15–20 repetitions were beneficial to achieve baseline levels (green line in Figure 2B). Training effectiveness increased substantially with the number of sets. Participants with a pre-COVID-19_legscore_ lower than ~2,500 kg reached their baseline levels after training session number 40, individuals with higher leg scores from the high TIG did not reach their baseline exercise performance, and most likely physical capacity, over the ~21 weeks until the second lockdown.

Regarding training intensity, it seems to be important to achieve momentary muscle fatigue during training by either a high number of repetitions or a higher training weight (33, 34), however, results are inconclusive. Based on the results of an umbrella review on the effectiveness of exercise interventions to increase muscle strength in the context of sarcopenia prevention, low-intensity resistance training [ ≤ 50% of the one repetition maximum (1RM)] may be sufficient to increase muscle strength, but high-intensity resistance training (~80% of the one repetition maximum) is recommended to optimize effects on strength (33, 35). Beckwée et al. (35) recommend 1–4 sets of 8–15 repetitions on 1–3 days a week. Based on our data, we recommend the following strategy to recover the pre-lockdown training level: training should be performed with at least 50 kg (sum of lifting and lowering) and 20 repetitions. However, individual training preferences in terms of low and high training weights should be considered and adjusted accordingly.

### 4.3. Strengths and limitations

This is the first study, to our knowledge, to explore the effect of closure of sports facilities during the first wave of the COVID-19 pandemic on physical performance, based on objective training data. Furthermore, the sample size of 17,450 individuals is large enough for sub-group analyses, e.g., different age groups and training intensity. As a limitation, we can only hypothesize that training intensity is a marker of physical fitness as we have no data on maximal strength, whether the weights were changed by the trainer or participant, whether the training was pushing the limits or not. As our data strengthen the importance of resistance training at a high intensity level, participants should be assessed for maximal training data or fitness data on a regular basis. Technically this could be integrated in the circuit. An according entry in the data base would be helpful for training monitoring as well as the use of training data in research. No further information on the study population is available concerning health issues, including infection with SARS-CoV-2, or individual exercise routines during the lockdown periods. Personal interviews to assess individual physical activity habits would offer important information for training recommendations. The study population is not representative for the population as a whole, as it is a selection of adults who exercise regularly in a sports facility.

### 4.4. Conclusion

The presented results examine objective training data of persons regularly exercising in a chip-controlled fitness circuit in Germany. In both, the middle-aged (45–64 yrs), and older age (≥65 yrs) groups, especially participants with vigorous training routines showed the greatest losses in exercise performance for upper and lower body muscle groups after the end of the first COVID-19-related lockdown, but no changes in endurance exercise performance. Endurance exercise, performed unrelated to the gym, seemed to have been effective in preserving individual training loads on the cycle ergometers. Independent of age, the high intensity training groups did not reach the pre-lockdown level, even after ~5 months of training. We conclude that a high intensity resistance training cannot be compensated without explicit resistance exercise. For the recovery of prior fitness levels, a training regimen of at least two sets with a minimum of 15 repetitions each and a training weight of ~50 kg was necessary in the analyzed study population. People, especially seniors, should be motivated to engage in more intense, but safe home-based resistance trainings to mitigate decreases in training loads, and eventually individual fitness, when sports facilities are not available, or individuals are hesitant to visit those facilities due to high incidences of infectious diseases. The use of health or fitness apps with focus on home-based resistance exercises may be a temporary alternative for training in gyms.

## Data availability statement

The data analyzed in this study is subject to the following licenses/restrictions: anonymous training data from a chip controlled training cycle. Requests to access these datasets should be directed to geriatrie@uol.de.

## Ethics statement

The study involving human participants were reviewed and approved by the Ethics Committee of the Medical Faculty of the University of Oldenburg. Written informed consent for participation was not required for this study in accordance with the national legislation and the institutional requirements.

## Author contributions

JK, SL, MH, and TZ developed the concept and design of the study. JK and TZ responsible for study management and prepared the manuscript. AS and FO-S conducted data clearance. FO-S conducted the statistical analysis. JK, TZ, and FO-S interpreted the data. All authors contributed to interpretation of data, drafting of the article, and final approval of the version to be published.
